# Impact of Dental Implant Surface Modifications on Osseointegration

**DOI:** 10.1155/2016/6285620

**Published:** 2016-07-11

**Authors:** Ralf Smeets, Bernd Stadlinger, Frank Schwarz, Benedicta Beck-Broichsitter, Ole Jung, Clarissa Precht, Frank Kloss, Alexander Gröbe, Max Heiland, Tobias Ebker

**Affiliations:** ^1^Department of Oral and Maxillofacial Surgery, University Medical Center Hamburg-Eppendorf, 20246 Hamburg, Germany; ^2^Department of Cranio-Maxillofacial and Oral Surgery, University of Zurich, 8032 Zurich, Switzerland; ^3^Department of Oral Surgery, Heinrich Heine University, 40225 Düsseldorf, Germany; ^4^Private Practice, 9900 Lienz, Austria

## Abstract

*Objective.* The aim of this paper is to review different surface modifications of dental implants and their effect on osseointegration. Common marketed as well as experimental surface modifications are discussed.* Discussion.* The major challenge for contemporary dental implantologists is to provide oral rehabilitation to patients with healthy bone conditions asking for rapid loading protocols or to patients with quantitatively or qualitatively compromised bone. These charging conditions require advances in implant surface design. The elucidation of bone healing physiology has driven investigators to engineer implant surfaces that closely mimic natural bone characteristics. This paper provides a comprehensive overview of surface modifications that beneficially alter the topography, hydrophilicity, and outer coating of dental implants in order to enhance osseointegration in healthy as well as in compromised bone. In the first part, this paper discusses dental implants that have been successfully used for a number of years focusing on sandblasting, acid-etching, and hydrophilic surface textures. Hereafter, new techniques like Discrete Crystalline Deposition, laser ablation, and surface coatings with proteins, drugs, or growth factors are presented.* Conclusion.* Major advancements have been made in developing novel surfaces of dental implants. These innovations set the stage for rehabilitating patients with high success and predictable survival rates even in challenging conditions.

## 1. Introduction

Nowadays, dental implants represent a reliable treatment option in oral rehabilitation of partially or fully edentulous patients in order to secure various kinds of prostheses. Dental implants have become a standard procedure for single tooth replacement in the esthetic zone, providing many advantages but also challenges in sophisticated patients.

Brånemark et al. first described the process of osseointegration more than 45 years ago [1, 2]. Their work launched a new era of research on shapes and materials of dental implants. But it was not until the last decade that the focus of biomedical research shifted from implant geometry to the osteoinductive potential of implant surfaces.

Today, roughly 1300 different implant systems exist varying in shape, dimension, bulk and surface material, thread design, implant-abutment connection, surface topography, surface chemistry, wettability, and surface modification [3]. The common implant shapes are cylindrical or tapered [4]. Surface characteristics like topography, wettability, and coatings contribute to the biological processes during osseointegration [5] by mediating the direct interaction to host osteoblasts in bone formation.

In general, the long-term survival rates of dental implants are excellent. However, implant failures still occur in a small quantity of patients. Primary implant failure due to insufficient osseointegration occurs in 1-2% of patients within the first few months [6]. Secondary implant failure develops several years after successful osseointegration in about 5% of patients and is commonly caused by peri-implantitis [6, 7]. The demographic trend in industrialized countries consecutively leads to an increase of elderly patients with advanced clinical conditions like impaired bone quality or quantity or other challenging comorbidities. Osseointegration might be impaired in patients with diabetes mellitus, osteoporosis, and comedication with bisphosphonates or following radiotherapy [8]. These patients remain a great challenge in dental implantology and prompt the need for bioactive surface modifications that accelerate osseointegration after implant insertion [8]. Besides, the aim of designing new bioactive surface properties is to accelerate osseointegration for more convenient, early loading protocols. The primary goal of biomedical research on surface modifications is to facilitate early osseointegration and to ensure a long-term bone-to-implant contact without substantial marginal bone loss.

In the first part of this paper, basic concepts of surface modifications are discussed and exemplified by major marketed implant types. In the second part, current experimental trends in implant surface modifications and their effect on osseointegration are depicted.

## 2. Review

### 2.1. Osseointegration of Dental Implants

Osseointegration of dental implants was previously characterized as a structural and functional connection between newly formed bone and the implant surface, which became a synonym for the biomechanical concept of secondary stability [9]. Osseointegration comprises a cascade of complex physiological mechanisms similar to direct fracture healing. The drilling of an implant cavity resembles a traumatic insult to bony tissue leading to distinct phases of wound healing [10]. Initially, mechanisms of cellular and plasmatic hemostasis lead to fibrin polymerization and the formation of a blood clot, which serves as a matrix for neoangiogenesis, extracellular matrix deposition, and invasion of bone forming cells [3, 11]. New bone generates from the borders of the drill hole (distance osteogenesis) or by osteogenic cells on the surface of the implant (contact osteogenesis). In distance osteogenesis, osteoblasts migrate to the surface of the implant cavity, differentiate, and lead to the formation of new bone. Thus, bone grows in an appositional manner towards the implant. In contact osteogenesis, osteogenic cells migrate directly onto the implant surface and generate* de novo* bone [3].

The secondary stability of a dental implant largely depends on the degree of new bone formation at the bone-to-implant interface [12]. According to Wolff's Law, the subsequent phase of load oriented bone remodeling leads to a replacement of primary woven bone to realigned lamellar bone in order to optimize the absorption of occlusal load [3, 11] and to transmit the mechanical stimuli to the adjacent bone [11]. At the end of the remodeling phase, about 60–70% of the implant surface is covered by bone [13]. This phenomenon has been termed bone-to-implant contact and is widely used in research to measure the degree of osseointegration [14]. According to the concept of mechanotransduction, bone remodeling continues lifelong [11]. Research efforts have been focused on designing novel topographies of implant surfaces to optimize osteoblastic migration, adhesion, proliferation, and differentiation.

### 2.2. Bulk Materials

The mechanical stability of a dental implant largely depends on the characteristics of its bulk material [15]. The core of the vast majority of dental implants is composed of titanium or titanium alloy due to the high biocompatibility and corrosion resistance as well as the favorable mechanical properties [5]. Today's growing demand for esthetic dental restorations has fueled the research on implants that mimic the color of natural teeth. Therefore, alternative nonmetal bulk materials, especially zirconium, become increasingly important [15]. A detailed discussion of different bulk materials is beyond the scope of this paper.

### 2.3. Modifications of Macrotopography

The surface topography of dental implants is crucial for adhesion and differentiation of osteoblasts during the initial phase of osseointegration as well as in long-term bone remodeling [3, 16]. Dental implant topography can be classified into macro-, micro-, and nanoscale. The macrotopography of an implant is determined by its visible geometry, for example, threads and tapered design. The metric scale is millimeters to micrometer. In recent years, scientific effort was mainly focused on micro- and nanogeometry. However, appropriate macrogeometry combined with adequate implant drill hole preparation is the fundamental basis of clinical success in dental implantology [17]. In theory, there are three basic concepts of bone healing pathways depending on the physical proximity at the bone-to-implant interface.

**Figure 1:**
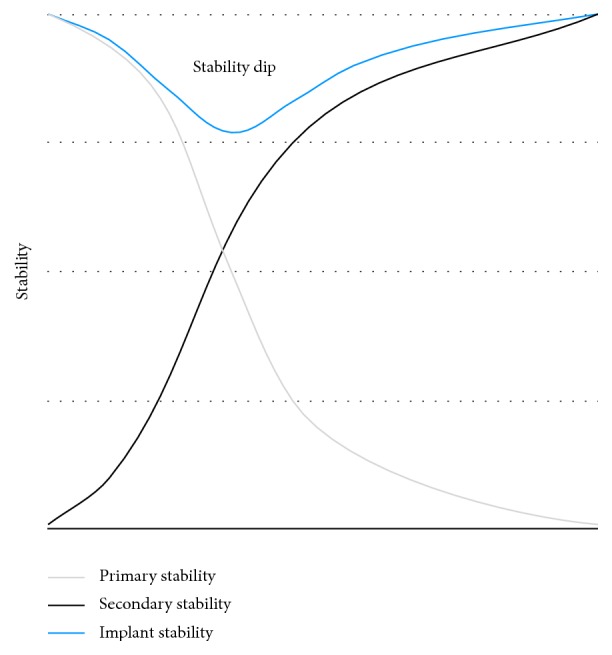
Mechanical stability of a dental implant after insertion. Primary stability decreases subsequently to implant insertion while secondary stability increases. After 2-3 weeks, the implant stability is the lowest in a phase called implant stability dip.

First, tight fit results when the diameter of the* inner* thread equals the dimensions of the socket, leading to potential microcracks of the surrounding bone. A high level of primary stability is initially achieved by friction. However, stability declines in the first weeks of bone healing due to compression necrosis of neighboring bone and subsequent bone remodeling, a process that has been previously described as implant stability dip (Figure 1) [17, 18]. Eventually, new bone is formed leading to secondary stability.

In the second scenario, the diameter of the* outer* thread is the same as the diameter of the implant cavity. The void space between the implant threads has been referred to as healing chambers [19]. These compartments ossify via formation of granulation tissue and contribute to osseointegration in secondary stability.

Third, the surgical instrumentation line lies right between the inner and the outer thread. In this case, regions of remodeling induced by compression and healing chambers coexist [17]. Healing chamber formation might be of significant importance for subsequent concepts of micro- and nanotopography, discussed hereafter, since migration of osteogenic cells requires void space [17].

### 2.4. Modifications of Microtopography

Until the 1990s, dental implants had primarily machined surfaces [20] which implies a turned, milled, or polished manufacturing process [4]. Imperfections along these machined surfaces enable osteogenic cells to attach and to deposit bone, thus generating a bone-to-implant interface. The healing time of machined implants is about 3 to 6 months depending on the anatomical location and the quality of bone [21]. Microtopography is linked to microroughness on a micrometer scale (1–100 *μ*m) and is modified by manufacturing techniques like machining, acid-etching, anodization, sandblasting, grit-blasting, and different coating procedures (Table 1) [5]. Commonly used scientific parameters to describe the surface roughness are the 2-dimensional *R*_*a*_ (profile roughness average) and the 3-dimensional *S*_*a*_ (area roughness average) [5]. The majority of dental implants on the market have a *R*_*a*_ of 1-2 *μ*m. According to Albrektsson and Wennerberg [22], this range seems to provide an optimal degree of roughness to promote osseointegration. Pits, grooves, and protrusions characterize the microtopography and set the stage for biological responses at the bone-to-implant interface. The modifications of microtopography contribute to an increase in surface area. Studies have shown increased levels of BIC for microrough surfaces [5, 23]. Changes in surface topography itself alter growth, metabolism, and migration as well as cytokine and growth factor production of osteogenic cells [21, 24]. The techniques of modifying the implant's microsurface are well-documented and have been clinical routine for decades.

#### 2.4.1. Sandblasted and Acid-Etched Implants

**Figure 2:**
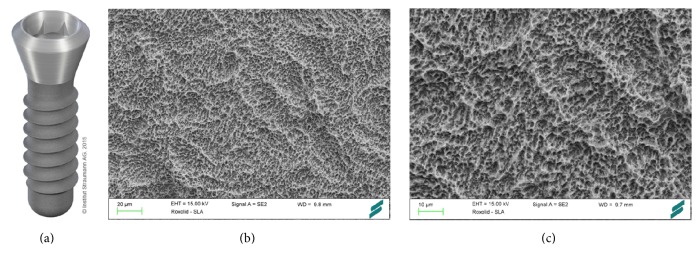
Roxolid implant with SLA surface (Straumann Holding AG, Basel, Switzerland). Roxolid dental implants (a) are made of titanium zirconium alloy. Large grit-blasting generates the macrolevel aspects of the surface (b), while the microtopographic features (c) are induced by acid-etching with HCl/H_2_SO_4_. Courtesy of Straumann Holding AG.

*Surface*. The macroroughness of the SLA (Sandblasted, Large grit, Acid-etched) (Straumann Holding AG, Basel, Switzerland) surface is manufactured by large grit sandblasting with 0.25–0.5 mm corundum particles at 5 bar [25]. The microtopographic surface structure is the result of a subsequent acid-etching process with HCl/H_2_SO_4_ at high temperatures [23] generating an active surface area with equal roughness and enhanced cell adhesion [21] (Figure 2). The Camlog Promote surface is based on a comparable approach (Camlog, Basel, Switzerland). The *S*_*a*_ value is 1.3 *μ*m and the surface topography is microrough [26].

*Preclinical Data.* The bone-to-implant contact of implants with different surface modifications was studied by Buser et al. [27] in a histomorphometric analysis in the miniature pig model. Three and 6 weeks after insertion, the SLA implants showed superior bone-to-implant contact (50–60%) compared to various other surface modifications as titanium plasma-sprayed (30–40%) or electropolished implants (20–25%). The sandblasting process also enhances the biomechanical features of acid-etched implants. Li et al. showed in a minipig model that SLA implants exhibit a superior bone anchorage compared to machined and acid-etched implants as removal torque values were significantly enhanced in SLA implants [28]. 

*Human Data.* In a prospective clinical trial by Fischer and Stenberg, 24 patients with edentulous maxillae were treated with full-arch prostheses on 139 SLA implants. The patients were followed up for 10 years [23]. This study showed satisfactory long-term results with an implant survival rate of 95.1% and a mean bone loss of 1.07 mm. Buser et al. [20] assessed the clinical outcomes of 511 SLA implants in 303 partially edentulous patients over a 10-year period in a retrospective study. The authors report a success rate of 97.0% and a 10-year implant survival rate of 98.8%. The rate of peri-implantitis was as low as 1.8%. In a multicenter study conducted by Cochran et al. [29], 385 SLA implants were placed in 120 patients in an early loading protocol. The 5-year success rate was 98.8% with a cumulative survival rate of 99.1%.

Similar survival rates have been published for Camlog implants. In a retrospective study, 40 edentulous patients who received 353 implants with the Camlog Promote surface were analyzed. A cumulative 4-year survival rate of 99.2% was observed [30].

#### 2.4.2. Grit-Blasted, Acid-Etched, and Neutralized Implants

**Figure 3:**
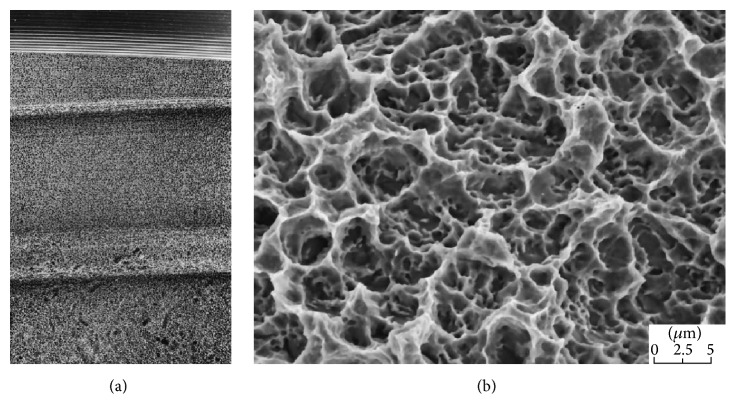
FRIADENT plus surface (DENTSPLY Implants, Mannheim, Germany). The surface of the FRIADENT plus surface (a) is created by large grit-blasting, etching, and a proprietary neutralizing technique. The hydrophilic surface features grooves that are interspersed with micropores (b). Courtesy of DENTSPLY Implants.

*Surface.* The FRIADENT plus surface (DENTSPLY Implants, Mannheim, Germany) has been adapted to DENTSPLY's ANKYLOS, XiVE, and FRIALIT implant systems. It is produced in a temperature controlled process by large grit-blasting (354–500 *μ*m), followed by etching in hydrochloric, sulfuric, hydrofluoric, and oxalic acid and finalized by a proprietary neutralizing technique [31]. The microtopography spans over several levels of magnitude and possesses a mean roughness of *R*_*a*_ = 3.19 *μ*m [31]. The macroroughness caused by grit-blasting is interspersed with irregularly shaped micropores. These micropores measure 2–5 *μ*m and contain a second level of even smaller sized micropores (Figure 3) [3]. The FRIADENT plus surface exerts dynamic changes in wettability. Upon contact to extracellular matrix proteins, the initial hydrophobic surface shifts to a hydrophilic state, exhibiting a water contact angle of 0° [31]. 

*Preclinical Data*. In a beagle dog model, Streckbein et al. [32] have compared the bone formation around four different implant types. The bone-to-implant contact was not significantly different after 6 and 12 weeks of healing for Brånemark MK III (Nobel Biocare Holding AG, Zürich, Switzerland), Osseotite (BIOMET 3i, Palm Beach Gardens, FL, USA), XiVE (DENTSPLY Implants, Mannheim, Germany), and Compress implants (IGfZ eG, Diez, Germany). The successful osseointegration of FRIADENT plus surfaced implants under the advanced clinical condition of immediate loading has been shown in a minipig model [33]. 85 dental implants were placed in the mandible and maxilla of 7 minipigs. After 4 months of healing, the immediately loaded implants exhibited an even higher degree of bone formation and remodeling compared to unloaded implants [33]. Novaes Jr. et al. [34] demonstrated in a dog model of periodontitis that FRIADENT plus surfaced implants, immediately placed in infected sites, reach a satisfactory bone-to-implant contact. 

*Human Data*. Clinical data on the FRIADENT plus surface is limited to a few studies. Degidi et al. [35] have compared 3 different DENTSPLY implant types with the FRIADENT plus surface in a clinical study on 321 patients. 802 implants were placed in an immediate or delayed loading protocol based on parameters of primary implant stability. One year after placement, the overall success rate was 99.6% with no significant difference between the 3 groups.

### 2.5. Modifications of Nanotopography

Biomechanical functioning* in vivo* spans from a visible scale to an atomic or nanometer scale. Nanotechnology has received wide attention in public and scientific media and its scale ranges from 1 to 100 nm. While the microtopography of the implant surface has been proposed to act at the cellular level of osseointegration [31], nanotopography of dental implants (Table 2) is thought to influence cell-implant interactions at the cellular and protein level. [36]. It was only years ago that biomedical engineers focused on the nanoscale of implant surface design [17]. Companies discovered that their implants exhibit aspects of nanotopography [25, 26]. An increase in surface energy is not merely a result of changes in surface roughness but is to a large extent caused by alterations of surface chemistry [17]. Thus, changes in nanotopography convey their effects at a physical, chemical, and biological level [3], resulting in increased adhesion of osteogenic cells [37] and thereby potentially promoting osseointegration. It has been hypothesized that the different osteoconductivity of micro- and nanoscale implant surfaces may influence osteoblast activity [11]. Further advancements in dental implant surface design are crucial to improve outcomes of sophisticated clinical situations as in immediate implantation after tooth extraction and early loading protocols and in patients with compromised bone or impaired wound healing capabilities [8].

#### 2.5.1. Discrete Crystalline Deposition (DCD)

**Figure 4:**
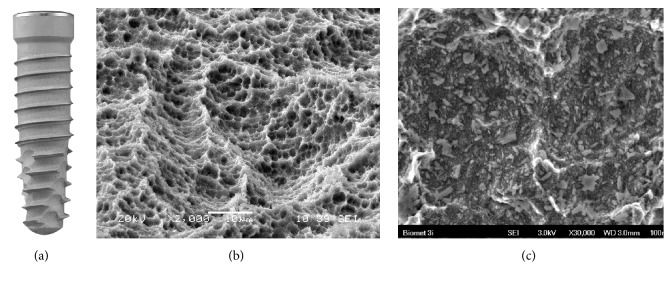
3i T3 (BIOMET 3i, Palm Beach Gardens, FL, USA). Small calcium phosphate particles are deposited on a double acid-etched surface in 3i dental implants (a). These particles are 20–100 nm (c) in size and form about half of the implant's total surface (b). Courtesy of BIOMET 3i.

*Surface*. In NanoTite and its successor the 3i T3 dental implant (BIOMET 3i, Palm Beach Gardens, FL, USA), the Osseotite surface (BIOMET 3i, Palm Beach Gardens, FL, USA) of the respective dual acid-etched titanium alloy implant has been altered with a nanometer scale manufacturing technique. Calcium phosphate (CaP) particles of 20–100 nm are deposited on a double acid-etched surface by a sol-gel process named Discrete Crystalline Deposition (DCD) (Figure 4). These CaP particles make up roughly 50% of the surface area [38] and exert a higher adhesive force to the implant surface than former techniques of CaP deposition [39, 40]. Bacterial adhesion to the NanoTite surface has been shown to be lower compared to the predecessor Osseotite surface [41]. 

*Preclinical Data.* Animal experiments have shown superior mechanical results of the DCD nanoscale surface. Mendes et al. [42] inserted titanium implants with the DCD surface and controls into the distal femur of rats. After 9 days* in vivo*, the disruption force at the bone-implant interface was significantly higher in DCD specimens compared to non-DCD samples. The same research group [43] reported increased osteoconduction of DCD treated implants compared to the predecessor control in a bone healing chamber model in rats. An animal study by Calvo-Guirado et al. [44] found only a tendency of increased bone-to-implant contact for DCD implants in a rabbit model.

*Human Data.* In a prospective 1-year clinical trial, 139 NanoTite tapered implants (BIOMET 3i, Palm Beach Gardens, FL, USA) were placed in 42 patients with a final torque of at least 30 Ncm in an immediate loading approach (20 single crowns, 30 fixed partial prostheses, and 7 full-arch maxillary reconstructions). 112 implants were inserted in the maxilla and 27 implants were placed in the mandible. The 1-year survival rate was 99.4% and the mean marginal bone resorption 1.01 mm [45]. A prospective, multicenter study with 335 NanoTite implants that were immediately provisionalized in 185 patients reported a 1-year survival rate of 94.9% [46]. Within the limits of these studies (short follow-up, no controls, and no randomization), these preliminary results are encouraging in providing an innovative implant nanotopographical surface for early loading protocols. The novel 3i T3 implant, the successor of the NanoTite implant with a similar DCD surface, has been marketed recently. However, limited clinical data is available, so far.

#### 2.5.2. Laser Ablation

**Figure 5:**
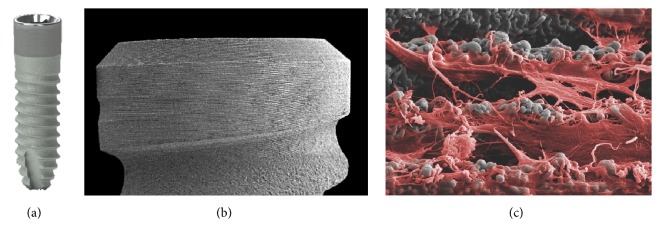
Laser-Lok implant (BioHorizons, Birmingham, AL, USA). A pattern of microchannels around the implant collar (b) is created by laser ablation. These cell-sized microchannels have been shown to act as a biological seal around the implant by fostering the attachment of connective tissue (c). Courtesy of BioHorizons IPH Inc.

*Surface.* An exception to the aforementioned is the Laser-Lok implant (BioHorizons, Birmingham, AL, USA) as its manufacturing technique focuses on improving the integration of dental implants in the surrounding soft tissue. Therefore, nanoscale surface manufacturing techniques have been transferred to the implant collar. The neck of the Laser-Lok implant has been processed in a laser micromachining step to generate a pattern of micro- and nanoscale microchannels (Figure 5). These microchannels have been proposed to act as a biologic seal by eliciting the attachment of connective tissue and bone and inhibiting epithelial downgrowth [47].

*Preclinical Data.* In a dog model, Nevins et al. [47] have demonstrated on histological specimens that connective tissue formation around Laser-Lok abutments is organized in a perpendicular manner. The dense cervical seal has been claimed to act like a barrier, thereby preventing apical migration of junctional epithelium.

*Human Data*. In a prospective, controlled clinical trial, 20 Laser-Lok implants were placed in 15 patients and clinical success parameters were evaluated. Control implants with a conventional machined collar were inserted neighboring the test implant. The mean probing depth was 2.3 mm for Laser-Lok implants versus 3.6 mm for control implants and the mean crestal bone loss was 0.59 mm for Laser-Lok implants compared to 1.94 mm for controls, suggesting the development of connective tissue around Laser-Lok implants [48]. Corresponding results have been published by different authors, demonstrating a beneficial influence of microtextured implant collars on soft tissue attachment and crestal bone preservation [49, 50]. A 2-year survival rate of 96.1% has been reported for Laser-Lok implants after immediate functional loading [51]. However, reports should be interpreted with care as long-term comparative studies are not available yet.

#### 2.5.3. Anodic Oxidation

**Figure 6:**
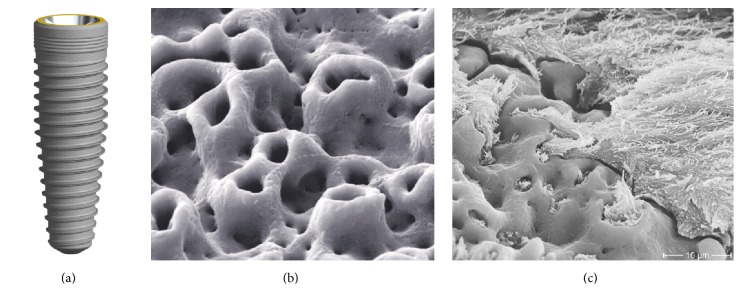
TiUnite surface (Nobel Biocare Holding AG, Zürich, Switzerland). The NobelReplace dental implant (a) is equipped with the TiUnite surface. The porous microstructure of the surface (b) has been suggested to promote osseointegration by providing additional retention in bone formation (c). Courtesy of Nobel Biocare.

*Surface.* A different technique of surface roughening has been applied to TiUnite (Nobel Biocare Holding AG, Zürich, Switzerland) implants. The implant surface is electrochemically modified by anodic oxidation to increase the thickness of the TiO_2_ layer from 17–200 nm in conventional titanium implants to 600–1000 nm (Figure 6). Thus, a porous surface microstructure with pore sizes of about 1.3–2.0 mm^2^, a porosity of roughly 20%, and a moderate degree of surface roughness of *S*_*a*_ = 1 *μ*m is generated [52]. Accordingly, this type of implant surface has also been referred to as titanium porous oxide (TPO) [53] or anodized titanium surface implant (ASI) [54]. In anodic oxidation, the implant is exposed to an electric circuit with the implant serving as an anode. TiUnite implants have been shown to possess nanoscale surface characteristics [55]. Besides, data from cell experiments suggest that anodic oxidation might be effectively transferred to the implant's neck in order to create a tight soft tissue seal. Nanostructured titanium surfaces generated by anodic oxidation have been shown to propagate adhesion, proliferation, and extracellular matrix deposition of human gingival fibroblasts [56].

*Preclinical Data.* Sul et al. [52] have shown in a rabbit model that the bone-to-implant contact is slightly greater in implants with anodized surfaces compared to commercially pure titanium implants. These data were substantiated by Zechner et al. [54] in a minipig model. The bone-to-implant contact of TiUnite implants was significantly greater compared to machined implants 6 and 12 weeks after implant placement. In this study, the TiUnite surface showed results comparable to those measured for HA-coated implant surfaces. In a Lekholm and Zarb type IV bone model conducted in monkeys, the bone-to-implant contact after 16 weeks of healing was reported to be 74%, thus suggesting a sufficient osteoconductive capacity in compromised bone sites [53].

*Human Data*. The beneficial biological responses to anodized titanium implant surfaces observed in animal studies were confirmed in clinical trials. Ivanoff et al. reported increased bone-to-implant contact of TiUnite microimplants compared to machined titanium microimplants. 20 patients received 1 test and 1 control implant. Histological samples were acquired 3 months after insertion in the mandible and 6 months after placement in the maxilla. A significantly greater BIC was measured around anodized implants in the mandibula and the maxilla [57]. In a clinical study of 394 implants inserted in 136 patients, the 5-month survival rate was 100% for TiUnite implants and 96.4% in the turned titanium control group [58]. The authors do not comment on statistical relevance. Despite the increased roughness of the anodized surface, the porous oxide surface does not facilitate enhanced biofilm formation [59].

#### 2.5.4. Titanium Oxide Blasted and Acid-Etched Implants

**Figure 7:**
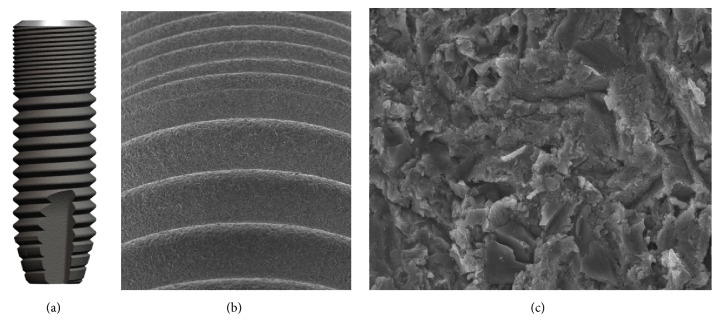
OsseoSpeed implant (DENTSPLY Implants, Mannheim, Germany). The nanolevel aspect (c) of the OsseoSpeed dental implant (a, b) is the result of titanium oxide blasting followed by etching with hydrofluoric acid. Accumulation of fluoride on the surface is a beneficial side effect of the manufacturing process. Courtesy of DENTSPLY Implants.

*Surface.* The OsseoSpeed implant (DENTSPLY Implants, Mannheim, Germany) was introduced to the market in 2004 [60]. The specific surface texture is a result of two subtractive, sequential manufacturing steps. Titanium oxide blasting produces the microscale surface roughness (Figure 7). The subsequent etching with hydrofluoric acid shapes the nanostructure of the implant [17]. A pleiotropic manufacturing effect is the accumulation of fluoride on the surface [61]. Fluoride containing surfaces have been hypothesized to propagate the host-to-implant reaction in early osseointegration [61]. Cell studies have demonstrated that the OsseoSpeed surface promotes a branched cell morphology of osteoblasts and an osteogenic gene expression profile as well as osteoinduction and osteogenesis in mesenchymal stem cells compared to TiOblast implants (DENTSPLY Implants, Mannheim, Germany), the titanium oxide blasted precursor [17].

*Preclinical Data.* Ellingsen et al. [62] have studied the biomechanical characteristics and the histomorphometric features of osseointegration in a rabbit model. For OsseoSpeed implants, significantly greater values of removal torque and shear strength as well as a higher degree of bone-to-implant contact were measured after 1 and 3 months of healing compared to unmodified controls [62]. In a healing chamber model, the amount of bone formation around OsseoSpeed implants was superior to the bone quantity around the precursor implant [17]. Comparison studies have been performed on OsseoSpeed implants. In a minipig model, OsseoSpeed implants and Straumann Bone Level Implants showed greater crestal bone preservation than NobelReplace Tapered Groovy Implants at 12 weeks after insertion [63]. Choi et al. have found a similar outcome of osseointegration in a rabbit model comparing OsseoSpeed implants to TiUnite implants [64].

*Human Data.* In a prospective, clinical trial, Mertens and Steveling [65] have investigated the long-term clinical outcome of OsseoSpeed implants. 42 implants in 15 patients were assessed over a 5-year period. The overall survival rate was 97% and the mean marginal bone loss 0.1 mm. These results were independent of immediate or conventional loading. In accordance, Raes et al. [66] have reported a 1-year survival rate of 98% in a prospective clinical trial on immediately provisionalized OsseoSpeed implants placed in the anterior maxilla of 48 patients. A 2-year prospective clinical trial by Collaert et al. [60] investigated the clinical outcome of 25 edentulous patients. Each patient was treated with 5 OsseoSpeed mandibular implants. The implants were provisionalized with a loaded screw retained restoration. The 2-year survival rate was 100% and a mean crestal bone loss of 0.11 mm was measured. In summary, the clinical studies available suggest a predictable overall outcome of OsseoSpeed dental implants. However, these results must be interpreted with care as none of the studies included a proper control group [17].

### 2.6. Surface Wettability

**Figure 8:**
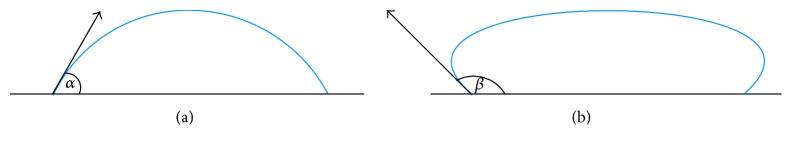
Concept of hydrophilicity. The hydrophilic surface on the left exhibits a water contact angle *α* < 90°, whereas the hydrophobic surface on the right shows a contact angle of *β* > 90°.

Besides topography and roughness, the surface wettability or hydrophilicity of implants is another central aspect of osseointegration. This chemical property is expressed by the water contact angle that ranges from 0° on very hydrophilic surfaces to greater than 90° on hydrophobic surfaces (Figure 8). Hydrophilic surfaces maintain the conformation and function of proteins whereas hydrophobic implant textures have been argued to trigger denaturation of proteins by exerting conformational changes [11]. The ability of cells to attach to and to migrate on the implant surface is driven by protein adsorption. Hydrophilic surfaces exert a higher affinity to proteins than hydrophobic surfaces [11]. Particular serum proteins possess cell binding domains, for example, arginine-glycine-aspartic acid (RGD) peptide, mediating subsequent cell attachment [11]. Besides, a high degree of hydrophilicity has been suggested to promote differentiation and maturation of osteoblasts, thereby contributing to an acceleration of osseointegration [67]. The use of dental implants with hydrophilic surfaces might prepone the onset of secondary stability.

#### 2.6.1. Hydrophilic Implants

**Figure 9:**
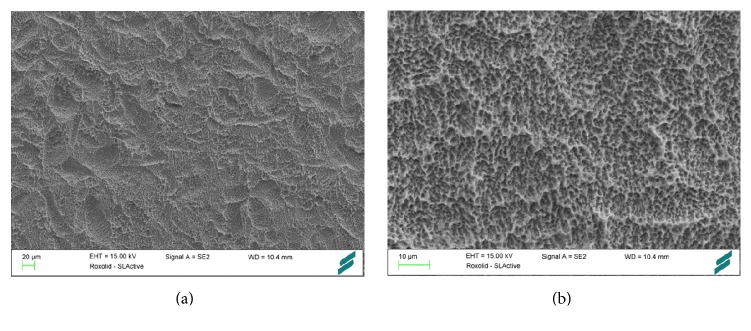
SLActive (Straumann Holding AG, Basel, Switzerland). In SLActive dental implants, the degree of hydrophilicity has been enhanced by rinsing under nitrogen protection and storage in saline solution. The SLActive surface (a, b) possesses elements of nanotopography. Courtesy of Straumann Holding AG.

*Surface*. The surface energy of conventional titanium oxide surfaces is low due to absorption of hydrocarbons and carbonates from ambient air and due to hydrophobicity resulting from surface roughness [67]. In SLActive dental implants (Straumann Holding AG, Basel, Switzerland), the standard large grit-blasted, acid-etched SLA implant surface has been modified to a high level of hydrophilicity [25] (Figure 9). The water contact angle of SLActive implants is 0° [16]. To prevent surface contact to air, SLActive implants are rinsed under nitrogen protection and stored in isotonic saline solution until insertion [67]. The high surface energy is sustained by a hydroxylated/hydrated surface that minimizes the absorption of contaminating hydrocarbons and carbonates from air [68]. Though not explicitly labeled as an implant with nano-surface structures, the SLActive implant exhibits elements of nanotopography [25, 26]. Alternatively, hydroxide ion solution may be applied to enhance the implant's surface wettability as demonstrated by Stadlinger et al. [69].

*Preclinical Data.* Biological responses to SLActive surfaces have been characterized in cell experiments. It has been claimed that the hydrophilic SLActive surface beneficially influences cell adhesion, stimulates maturation of osteogenic cells, promotes a bone forming microenvironment, and fosters neoangiogenesis [25]. Schwarz et al. [70] have studied the histological differences in osseointegration of SLActive implants compared to SLA implants in a dog model. For SLActive implants, a higher affinity of the initial blood clot to the implant surface, an enhanced neoangiogenesis, increased bone-to-implant contact, and greater bone density were described within the first 2 weeks of bone healing [70]. Buser et al. confirmed a higher BIC for SLActive compared to SLA implants 2 and 4 weeks after implant placement but not after 8 weeks, strengthening the theory that hydrophilic surfaces are beneficial in early phases of osseointegration [71]. Accordingly, significantly greater removal torque values were measured for SLActive implants as opposed to SLA implants, suggesting a superior bone anchorage in early implant healing [72].

In a minipig model, the bone formation around implants modified by hydroxide ions (SPI Element, Thommen Medical AG, Waldenburg, Switzerland) was tested. No clear differences to the control implants were found. However, there was a trend towards an increased BIC early after implant placement [69]. Other authors have substantiated these findings, showing an increased bone formation around hydroxide ion-treated dental implants. In a dog model using immediately inserted implants, Calvo-Guirado et al. have shown an increased BIC and less crestal bone resorption for hydrophilic implants 12 weeks after implant placement [73].

*Human Data*. Randomized, controlled clinical trials on SLActive implants are still scarce [25]. On review of the available human studies, Wennerberg et al. [25] have found little clinical evidence so far to clearly state a preference for SLActive over SLA implants. In a split-mouth study, SLActive implants were compared to SLA implants with early loading protocols in irradiated patients. 102 implants were placed in 20 patients in both jaws. At 1-year follow-up, there was a high survival rate (100% for SLActive versus 96% for SLA implants) and low crestal bone loss <0.4 mm in both groups with no significant difference. Accordingly, both implants types were found to be suitable for early loading protocols in irradiated patients [74]. In a RCT, Ti Grade IV and TiZr small-diameter implants with SLActive surface were investigated. Comparable survival rates of 97.8% (Ti Grade IV) and 98.9% (TiZr) and similar success rates of 94.4% (Ti Grade IV) and 96.6% (TiZr) were measured after 1 year with no significant difference between the two implant materials with a SLActive surface [75]. Further studies reported high success rates for SLActive implants in early loading without [76] and with full occlusion [77] and acceptable survival rates of 95.7% after 1 year and 92.3% after 2 years for 4-mm short implants [78].

In summary, despite promising preclinical data, convincing reports demonstrating a clear clinical superiority of SLActive over SLA implants are so far nonexisting.

### 2.7. Photofunctionalization

**Figure 10:**
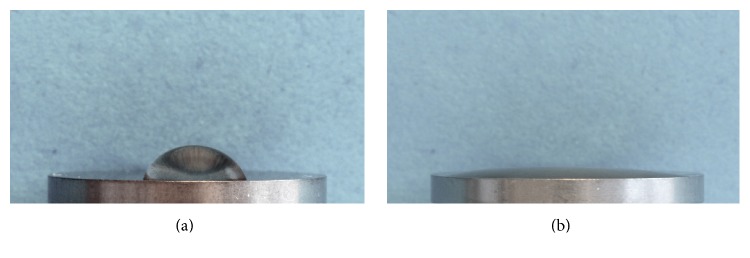
Hydrophilic effects of UV treatment. Pure titanium discs were subjected to photofunctionalization using UV light. Droplets of water (20 *μ*L) were placed on untreated (a) and photofunctionalized discs (b). The water contact angle is drastically decreased by UV treatment (b), illustrating the hydrophilic effects of photofunctionalization. Courtesy of Henningsen.

UV treatment of dental implant surfaces enhances bioactivity and osseointegration by altering the titanium dioxide on the surface. By promoting interactions of cells and proteins to the implant on a molecular level, UV light is believed to enhance the osteoconductivity [79]. UV treatment reduces the degree of surface hydrocarbon and increases surface energy and wettability (Figure 10) [80–83]. UV light has been suggested to raise the level of protein absorption and cellular attachment to titanium surfaces and has been shown to restore bioactivity caused by age-related degradations [84, 85].

*Preclinical Data.* In a dog model, Hirakawa et al. [86] investigated the effect of photocatalytic wettability induced by UV-A irradiation on osseointegration of dental implants. Bone-to-implant contact was significantly enhanced in UV-A irradiated titanium implants after 2 weeks of healing but not after 4 weeks. The authors conclude that UV-A treatment of titanium implants accelerates bone formation particularly in early phases of osseointegration. Corresponding results have been published by Park et al. in a rabbit tibia model [81]. UV-C irradiated anodized titanium implants showed a significantly higher bone-to-implant contact and amount of bone compared to control implants after 4 weeks of healing. After 12 weeks of healing, no significant differences were observed. Similar findings have been reported by Aita et al. in a rodent model [85]. UV pretreatment of machined as well as acid-etched titanium implants fostered attachment, proliferation, and differentiation of osteoblasts. Higher degrees of bone formation in the UV treated groups translated into improved biomechanical properties in push-in tests [85].

*Human Data.* Clinical data on photofunctionalized dental implants is limited to 2 publications. In a case series of 7 implants placed in 4 patients with compromised bone, Funato and Ogawa claimed a significant gain in marginal bone levels after 1 year and a considerable increase in implant stability quotient of photofunctionalized implants [87]. In a retrospective analysis, 70 patients received photofunctionalized implants with a mainly small-diameter configuration in early loading protocols. The authors reported a high success rate of 97.6% [83].

In summary, available data indicate that UV treatment restores and even improves the bioactivity of titanium implants, thereby enhancing the degree of bone formation particularly in early phases of osseointegration.

### 2.8. Future Perspective: Surface Coatings

In order to meet the challenges of advanced indications in dental implantology, tremendous scientific effort is currently focused on bioactive surface coatings. The basis of this field of research is the genuine biological character of osseointegration. These innovative approaches intend to mimic the biochemical milieu and nanostructural architecture of human bone. Coatings comprise specific agents, drugs, proteins, or growth factors. The clinical goals of biomaterial research have been (1) the optimization of implant stability by interacting with natural cascades of osseointegration, (2) the improvement of peri-implant soft tissue integration, and (3) the reduction of peri-implantitis by impairing bacterial adhesion to the implant surface. Prerequisite of any surface coating is its resistance against disintegration during insertion [88]. The following section provides an overview of recent innovations in this field, didactically based on the substance's order of appearance in osseointegration.

#### 2.8.1. Hydroxyapatite and Nanocomposite Coatings

Bone consists of cells (osteocytes, osteoblasts, and osteoclasts) and bone matrix. The bone matrix is made of water and a composite of organic and inorganic components. Constituents of the organic matrix are proteins [89]. Collagen type I accounts for up to 90% of these proteins. Among others are collagen type V, osteocalcin, osteopontin, osteonectin, and fibronectin. Hydroxyapatite (HA) is a very stable biological form of CaP and strengthens the organic matrix by mineralization [90]. Biomimetic surface techniques attempt to promote osseointegration by integration of a singular component or a combination of these elements into the implant surface [91].

HA coatings resemble a reservoir of calcium and phosphate [91] in addition to their biomimetic property. For several years, titanium plasma spraying was the commonly applied technique to deposit CaP on implant surfaces [92]. A powder was dispersed into a plasma torch that is targeted on the implant resulting in a CaP thickness of 40–50 *μ*m [21]. Uncertainty exists regarding the long-term stability of plasma-sprayed HA coatings [93] and long-term clinical outcomes were poor [94].

A recently introduced surface treatment generates a hydrophilic monolayer of multiphosphonic acid molecules on the outside of the implant surface, thus imitating natural hydroxyapatite (SurfLink, Nano Bridging Molecules, Gland, Switzerland) [95]. In a sheep model, multiphosphonate treated implants exhibited significantly greater biomechanical stability compared to untreated controls [96]. Clinical data on SurfLink implants is scarce, so far. In a study on 32 patients using a slit-mouth design, the 1-year survival rate was 100% and the mean marginal bone level change −0.27 mm with no significant difference to untreated control implants [95].

To imitate the biological environment of nanoscale crystals in native bone tissue, nanotechnology has become of pivotal importance to compose nanoscale hydroxyapatite- (nHA-) containing implant surfaces. Extensive work has been carried out to transfer nanotechnology to HA coatings. As mentioned earlier in this paper, DCD is a well-documented and reliable technique to attach nanoscale CaP particles to the implant surface [38]. nHA is used as a single compound coating or as part of a composite in combination with carbon nanotubes, collagen, titanium dioxide, bioglass, silica, or ceramic oxide [91]. A major advantage of nanocomposites is the ability to adjust the mechanical characteristics of the implant to those of natural bone, for example, to avoid negative stress shielding [91].

#### 2.8.2. Growth Factors

In hemostasis, the first phase of osseointegration, platelets, which have been liberated to the alveolar bone from damaged vessels, degranulate and release specific growth factors that initiate the second phase of osseointegration, the inflammatory phase. These factors comprise platelet-derived growth factor (PDGF), transforming growth factor beta (TGF-*β*), and fibroblast growth factor (FGF) [11]. Macrophages resemble a second important source of growth factors. Upon elimination of cell detritus, these cells release VEGF (vascular endothelial growth factor), PDGF, and FGF to initiate the proliferative phase of osseointegration [11]. VEGF induces neoangiogenesis that is crucial for osteogenesis [11].

Bone morphogenetic proteins were first described in 1965 and comprise a group of at least 18 growth factors that belong to the TGF-*β* family [3].* In vivo*, BMPs are released from osteoblasts, platelets, and endothelial cells and are deposited into the bone matrix until being liberated during socket preparation [89]. BMPs regulate genes for collagen, alkaline phosphatase, and osteopontin [89]. BMP2, BMP4, and BMP7 exclusively stimulate bone formation [89]. To acquire an adequate yield of BMPs, these proteins have to be produced in a recombinant technique [89].

BMP2-containing biomimetic CaP coatings on a titanium disk led to sustained ectopic ossification in a rat model [97]. In a pig calvaria model, BMP2-bearing CaP coatings enhanced bone density but not overall BIC when compared to acid-etched controls [98]. Susin et al. [99] have shown in a supra-alveolar, critical-size defect model in dogs that recombinant BMP7-coated dental implants induce relevant vertical augmentation of the alveolar ridge. The effect of BMP2- and VEGF-coated implants was investigated in a bone defect model in dogs [100]. Compared to anodized control implants, a significantly enhanced bone-to-implant contact and increased new bone formation were detected in the BMP2 and the BMP2 plus VEGF group after 8 weeks of healing. These 2 groups showed comparable results. The application of TGF-*β* to the implant surface has been studied by De Ranieri et al. in a rat model [101]. TGF-*β* significantly enhanced the bone-to-implant contact and the bone volume around implants that were placed in the rodent femur. FGF-2 influences the proliferation of osteoblasts and has been studied as an implant coating. FGF-2-bearing nanoparticles were coated on titanium implants and enhanced osseointegration in a rabbit tibia model [102]. Encouraging preclinical data has also been published for PDGF. Implants that were coated with recombinant PDGF exhibited enhanced osteogenic differentiation and proliferation* in vitro* and improved osseointegration compared to control titanium implants in osteoporotic rats [103].

#### 2.8.3. Extracellular Matrix Proteins

In the proliferative phase of osseointegration, fibroblasts are triggered by FGF to secrete extracellular matrix proteins like collagen, chondroitin sulfate, fibronectin, vitronectin, and other proteoglycans [11]. The extracellular matrix provides crucial guidance for osteoprogenitor cells that migrate to the implant via interaction of integrins on the cell surface and RGD motifs of fibronectin [11]. Osteoblasts have been proposed to originate from a subset of mesenchymal stem cells that line minor vessels and are known as pericytes [104]. Upon the release of BMP, these cells differentiate into osteoblasts [11].

Dental implants coated with extracellular matrix proteins have shown a positive effect on peri-implant bone formation in preclinical studies. de Barros et al. [105] reported an increase in bone volume and mineralization for collagen type II/chondroitin sulfate coated implants compared to uncoated controls in a dog model. In contrast, Korn et al. [106] found no significant difference in BIC 4 weeks after implant placement of collagen/chondroitin sulfate coated or collagen/sulfated hyaluronan coated implants compared to grit-blasted, acid-etched implants.

#### 2.8.4. Peptides

Peptides are biomolecules composed of short sequences of amino acids. They resemble fragments of larger proteins. Particular peptides that facilitate cell adhesion in osseointegration or that exert antibacterial effects have been employed to design novel implant surfaces. The RGD peptide is an important sequence of extracellular matrix proteins that acts as a binding site for integrin receptors in adhesion and migration of osteogenic cells [10]. The clinical significance of RGD peptide coatings is uncertain, so far. Schliephake et al. [107] have investigated the effect of cyclic RGD peptide coatings on osseointegration in a dog model. After 4 weeks, implants with RGD peptide/collagen I coating showed a significantly higher degree of BIC compared to machined titanium implants. The impact of pure RGD peptide coatings seems to be comparable to other organic coatings as implants coated with collagen or chondroitin sulfate show similar histomorphometric results [108]. Broggini et al. reported no significant effect of RGD peptide coatings in a minipig model compared to SLActive control implants [109].

Besides, the coating of dental implants with antibacterial peptides has been investigated. There is general consent that peri-implantitis is the main cause of long-term implant failure [7]. Recently, several innovative attempts have been made to equip the surface of dental implants with bactericidal properties. Research in this field is quite young and reports cover primarily preclinical experiments. GL13K has been derived from a defense protein found in saliva and has proven biocompatibility and antibacterial function on titanium discs in a* Porphyromonas gingivalis* model [110]. Human beta defensins (HBDs) are peptides that convey antibacterial effects on epithelial borders. In cell experiments, HBDs exhibited biocompatibility and were able to promote proliferation of osteoblasts and mesenchymal stem cells [111].

#### 2.8.5. Messenger Molecules

The remodeling phase succeeds the proliferative phase. Woven bone is transformed into load oriented trabecular bone [11]. In bone remodeling, osteoblasts interact closely with osteoclasts. Sclerostin is one of the messenger molecules that mediates the osteoblast-osteoclast interaction. It is secreted by osteocytes and serves as an inhibitor of osteogenesis by blocking osteoblastic bone formation [112]. Systemic administration of antibodies that block the physiologic effects of sclerostin improved bone anchorage of titanium implants in a rat model of osteoporosis [113]. Studies on coatings with sclerostin antibodies are not available yet. However, antisclerostin coatings might pose a promising tool to enhance osseointegration of dental implants.

#### 2.8.6. Drug Coatings

HA coatings have been successfully used as local drug delivery systems. Statins inhibit the HMG-CoA reductase and are prescribed in dyslipidemia. When incorporated in the implant surface, statins have been claimed to trigger the local liberation of BMPs, thus promoting osseointegration [114].

Bisphosphonates are antiresorptive drugs that influence bone metabolism mainly by inhibition of osteoclasts [115]. Common indications include metastatic bone disease or osteoporosis [115, 116]. Peter et al. [117] demonstrated in a rat model that implants with a Zolendronate containing HA coating yield a higher peri-implant bone density and promote increased mechanical fixation. In an osteoporotic rat model, Stadlinger et al. demonstrated increased BIC and a higher level of bone mineralization of Zolendronate loaded implants [114]. In a randomized clinical trial on 16 patients, dental implants with a bisphosphonate-eluting fibrinogen coating showed a significantly enhanced mechanical fixation, measured by resonance frequency analysis [118].

## 3. Conclusion

Numerous preclinical studies have shown the superiority of particular surface modifications in respect to histomorphometric properties and biomechanical features. However, human studies translating these preclinical data into superior clinical performance when comparing certain types of implants are scarce. The central focus of implant development is to minimize bacterial adhesion while promoting recruitment, adhesion, and proliferation of osteogenic as well as fibroblastic cells in order to gain a high degree of hard and soft tissue integration. To guarantee long-term success in clinically challenging conditions, the development of multifunctional surface modifications and coatings is necessary. The goal of future research is to design a single polyvalent implant type with enhanced clinical behavior in regard to osseous and fibrous integration and prevention of peri-implantitis.

## Figures and Tables

**Table 1:** Examples of dental implants with microtopographical surface features.

Sandblasted and acid-etched implants	Grit-blasted, acid-etched, and neutralized implants
(i) SLA, Straumann (ii) Camlog Promote surface	(i) FRIADENT plus surface, DENTSPLY

**Table 2:** Examples of dental implants with nanotopographical surface characteristics.

Discrete Crystalline Deposition (DCD)	Laser ablation	Anodic oxidation	Titanium oxide blasted and acid-etched implants	Hydrophilic implants
(i) NanoTite/T3, BIOMET 3i	(i) Laser-Lok, BioHorizons	(i) TiUnite, Nobel Biocare	(i) OsseoSpeed, DENTSPLY	(i) SLActive, Straumann
